# The new variant c.458C>*G* in CHRNA1 causes late-onset post-synaptic slow-channel congenital myasthenic syndrome

**DOI:** 10.1016/j.clinsp.2025.100791

**Published:** 2025-09-24

**Authors:** Josef Finsterer, Fulvio A. Scorza, Carla A. Scorza, Ana C. Fiorini

**Affiliations:** a Neurology Dpt., Neurology & Neurophysiology Center, Vienna, Austria; b Universidade Federal de São Paulo (UNIFESP/EPM), São Paulo, SP, Brazil; c Department of Speech Therapy, Escola Paulista de Medicina, Universidade Federal de São Paulo (EPM/UNIFESP), São Paulo, SP, Brazil; d Postgraduate Studies Program in Speech Therapy, Pontifícia Universidade Católica de São Paulo (PUC-SP), São Paulo, SP, Brazil

## Abstract

• Avoid pyridostigmine in slow channel syndrome due to CHRNA1 variants.
• Congenital myasthenic syndrome due to CHRNA1 mutations may have a late onset.
• Salbutamol can be beneficial in carriers of CHRNA1 variants.

## Correspondence

Congenital Myasthenic Syndromes (CMS) are a genetically and phenotypically heterogeneous group of inherited diseases that are caused by a defect in neuromuscular transmission.^1^, ^2^, ^3^ Mutated proteins in the presynaptic or postsynaptic membrane, in the synaptic cleft or under the postsynaptic membrane are responsible for the phenotype, which consists of developmental delay, mental retardation, laryngeal, pharyngeal, axial, respiratory and limb muscle weakness, manifesting as ptosis, ophthalmoparesis, dysarthria, dysphagia, respiratory insufficiency, falling head syndrome or quadruparesis, hypotonia and arthrogryposis.^1^, ^2^, ^3^, ^4^ One of the proteins in the postsynaptic membrane that is frequently mutated in CMS is CHRNA1.^1^, ^2^, ^3^ Depending on the defect in acetylcholine receptor kinetics, CHRNA1 mutations lead to either slow channel syndrome (autosomal dominant inheritance) or fast channel syndrome (recessive inheritance).^1^, ^2^, ^3^, ^4^ A patient with slow-channel CMS due to the c.458C>*G* mutation in CHRNA1 has not yet been reported.

The patient is a 53-year-old woman who was diagnosed with CMS at the age of 43 after a sudden respiratory arrest followed by cardiac arrest. Prior to the respiratory arrest, she had developed generalized fatigue and a bowed head for two years (Table 1). She was successfully resuscitated and thoroughly investigated for the cause of the respiratory arrest and secondary cardiac arrest. Low-frequency Repetitive Nerve Stimulation (RNS) indicated a postsynaptic defect and genetic testing revealed the heterozygous variant c.458C>*G* (T153S) in CHRNA1. She was not treated until she was 43-years-old, when salbutamol 4 mg/d was started. Salbutamol improved general fatigue and respiratory muscle weakness within six months and the quantitative myasthenia gravis score improved from 27 to 0. In addition, she received furosemide and sorbisterite for hyperkalemia. A brief trial of pyridostigmine worsened her condition (Table 1). Since then, she has developed muscle weakness, mainly affecting the neck and back muscles and, more recently, the left hand. Speaking, swallowing and biting were not affected. She felt generally weak and easily fatigued. Her daughter is also a carrier of the variant in the heterozygous form, but has not yet developed CMS symptoms. The patient required non-invasive CPAP ventilation at night.

**Table 1:** Timeline of symptoms.

Symptoms	Intervention	Outcome
Generalised fatigue and easy fatigability at age 41	None	Persisted
Respiratory failure at age 43	Resuscitation, salbutamol	Improvement of fatigue
Weakness of neck and back muscles	Pyridostigmine	Deteriorated muscle weakness
Nightly desaturations	CPAP	Improved oxygenation
Weakness of the left hand	3,4-DAP	Improved hand weakness

The patient presented is of interest for several reasons. First, CMS was due to a novel mutation in CHRNA1. Extensive searches for an earlier description in ClinVar were negative. Whether this variant was truly causative remains speculative, but in the absence of an alternative explanation, it is very likely that the discovered variant was indeed responsible for the phenotype. The variant most likely developed de novo in the index patient, as none of her predecessors were clinically affected, but parental testing was unavailable. Secondly, the clinical manifestations did not occur until the age of 41, indicating a late onset of CMS. Late-onset forms of CMS have been described previously and occur not only in cases with CHRNA1 mutations, ,^5^ but also in carriers of GLPT1 mutations,^6^ DOK1^7^ or MUSK.^8^ Third, the patient benefited from salbutamol over a ten-year period, but as the disease progressed, she required additional medication such as 3,4-DAP. A short trial of pyridostigmine worsened her symptoms. Worsening of symptoms under pyridostigmine has been previously described and occurs in some patients with slow-channel syndrome.^1^ Fourth, the patient had hyperkalemia, which has not previously been reported in patients with CHRNA1 mutations and, therefore, most likely is incidental. Patients carrying CHRNA1 variants other than the one in the index patient presented with easy fatigability, facial dysmorphism, facial weakness, or limb weakness.^9^^,^^10^ The fifth issue is that the diagnosis was delayed by two years, which put her at risk of receiving medications that could have exacerbated her symptoms. Fortunately, during the period when the diagnosis was not yet known, no medication was administered that could have worsened the symptoms.

In summary, this case demonstrates that mutations in CHRNA1 causing post-synaptic slow channel CMS can have a late clinical onset, respond to salbutamol, but require additional medication in the form of 3,4DAP, fluoxetine or quinidine over time.

### Statement of ethics

a) The study was approved by the institutional review board (responsible: Finsterer J.) at the 4th November 2023. b) Written informed consent was obtained from the patient for publication of the details of their medical case and any accompanying images.

## Data availability

Data that support the findings of the study are available from the corresponding author.

## Compliance with ethics guidelines

This article is based on previously conducted studies and does not contain any new studies with human participants or animals performed by any of the authors.

## Authors’ contributions

JF: Design, literature search, discussion, first draft, critical comments, final approval.

## Funding

No funding was received.

## Declaration of competing interest

The author declares that the research was conducted in the absence of any commercial or financial relationships that could be construed as a potential conflict of interest.
